# Treatment of Asthenic Dropsy, by the Preparations of Nux Vomica

**Published:** 1852-05

**Authors:** 


From the Gazette Med. de Paris.

*Treatment of Asthenic Dropsy by the Preparations of Nux Vomica.* By TESSIER.

SEROUS infiltration of paralyzed limbs is dissipated with the paralysis; and paralysis is sometimes cured by nux vomica. This medicine does not act alone on the nerves of the life of relation, but also on those of organic life, since it causes the intestines to contract, and hence constitutes an excellent remedy for constipation caused by inertia of the digestive tube. Could not the same medicine be made to act upon the absorbent vessels, and communicate to them greater activity, and thus cause the resorption of those effusions connected with a general or local asthenia? This question was proposed by M. Tessier, and his experience, seconded by other authority, has decided the question affirmatively.

Five cases of this kind have been reported by M. Tessier, all of which are significant and conclusive on the subject. In the first, there was œdema of the lower limbs, succeeding a case of diabetes, cured by liquid ammonia. At the end of a month, there existed no trace of swelling, but the diabetes returned. The treatment by ammonia was resumed, and the diabetes again disappeared; then the œdema returned, which was once more treated by nux vomica, and was the second time cured. The second was the case of an individual much enfeebled by imperfect nutrition, and suffering from considerable œdema of the lower extremities, and incipient ascites. Nux vomica was employed, and at the end of eight days there was evident improvement; and by the 25th day the cure was complete. The third was the case of a military man, affected with ascites and œdema of the inferior extremities, the result of an intermittent fever; both quinine and ferruginous preparations had failed. The nux vomica was then administered, and the cure was so far advanced at the end of eight days, that the patient regarded himself cured, and left the hospital. Finally, says M. Tessier, the fourth case was one of œdema of the legs, succeeding an attack of typhoid fever, which left the patient greatly enfeebled, but which was cured in five days. He gave the nux vomica in doses of 2 and 5 *centigrammes* daily.

It is scarcely necessary to remark, that M. Tessier relies upon this medicine only in purely asthenic dropsy. Of course it must fail when any material obstacle is offered to the passage of the blood through the venous trunks.

